# Transitions from Aerobic to Anaerobic Metabolism and Oxygen Debt during Elective Major and Emergency Non-Cardiac Surgery

**DOI:** 10.3390/biomedicines12081754

**Published:** 2024-08-05

**Authors:** Nikolaos Papagiannakis, Dimitrios Ragias, Nicoleta Ntalarizou, Eleni Laou, Aikaterini Kyriakaki, Theodoros Mavridis, Amir Vahedian-Azimi, Minas Sakellakis, Athanasios Chalkias

**Affiliations:** 1 First Department of Neurology, Eginition University Hospital, Medical School, National and Kapodistrian University of Athens, 11528 Athens, Greece; nikolas.papagia@gmail.com; 2 Medical Center of Sofades, General Hospital of Karditsa, 43100 Karditsa, Greece; mjr9898@hotmail.gr; 3 Postgraduate Study Program (MSc) “Resuscitation”, School of Medicine, National and Kapodistrian University of Athens, 11527 Athens, Greece; nntalarizou@gmail.com; 4 Department of Anesthesiology, Agia Sophia Children’s Hospital, 11527 Athens, Greece; elenilaou1@gmail.com; 5 Department of Anesthesiology, General Hospital of Syros Vardakeio and Proio, 84100 Syros, Greece; katrinkyr@hotmail.com; 6 Department of Neurology, Tallaght University Hospital (TUH)/The Adelaide and Meath Hospital Incorporating the National Children’s Hospital (AMNCH), D24 NR0A Dublin, Ireland; mavridismdr@yahoo.gr; 7 Nursing Care Research Center, Clinical Sciences Institute, Nursing Faculty, Baqiyatallah University of Medical Sciences, Tehran 1435915371, Iran; amirvahedian63@gmail.com; 8 Department of Medicine, Jacobi Medical Center-North Central Bronx Hospital, Bronx, NY 10467, USA; doctorsakellakis@gmail.com; 9 Institute for Translational Medicine and Therapeutics, University of Pennsylvania Perelman School of Medicine, Philadelphia, PA 19104-5158, USA; 10 Outcomes Research Consortium, Cleveland, OH 44195, USA

**Keywords:** oxygen transport, oxygen debt, aerobic metabolism, anaerobic metabolism, cardiovascular dynamics, hemodynamics, hemodynamic coherence, microcirculation, major non-cardiac surgery, emergency surgery

## Abstract

Introduction: Intraoperative hemodynamic and metabolic optimization of both the high-risk surgical patients and critically ill patients remains challenging. Reductions in oxygen delivery or increases in oxygen consumption can initiate complex cellular processes precipitating oxygen debt (OXD). Methods: This study tested the hypothesis that intraoperative changes in sublingual microcirculatory flow reflect clinically relevant transitions from aerobic to anaerobic metabolism (TRANAM). We included patients undergoing elective major and emergency non-cardiac surgery. Macro- and microcirculatory variables, oxygen extraction, and transitions of metabolism were assessed in both cohorts. Results: In the elective group, OXD was progressively increased over time, with an estimated 2.24 unit increase every 30 min (adjusted *p* < 0.001). Also, OXD was negatively correlated with central venous pressure (ρ = −0.247, adjusted *p* = 0.006) and positively correlated with stroke volume variation (ρ = 0.185, adjusted *p* = 0.041). However, it was not significantly correlated with sublingual microcirculation variables. In the emergency surgery group, OXD increased during the first two intraoperative hours and then gradually decreased until the end of surgery. In that cohort, OXD was positively correlated with diastolic arterial pressure (ρ = 0.338, adjpatients and the critically ill patients remains challengingsted *p* = 0.015). Also, OXD was negatively correlated with cardiac index (ρ = −0.352, adjusted *p* = 0.003), Consensus Proportion of Perfused Vessels (PPV) (ρ = −0.438, adjusted *p* < 0.001), and Consensus PPV (small) (ρ = −0.434, adjusted *p* < 0.001). Conclusions: TRANAM were evident in both the elective major and emergency non-cardiac surgery cohorts independent of underlying alterations in the sublingual microcirculation.

## 1. Introduction

Intraoperative hemodynamic and metabolic optimization of both the high-risk surgical patients and the critically ill patients remains challenging. Despite the progress in anesthesiology and critical care medicine and the development of sophisticated monitoring techniques, inadequate organ perfusion with resultant metabolic derangements represents a common pathway for postoperative morbidity and mortality. Notably, the detection of perfusion abnormalities using global hemodynamic parameters is not always possible as systemic hemodynamics do not consistently reflect effective microcirculatory perfusion and oxygen delivery (DO_2_) to the parenchymal cells [1]. Moreover, physiological intra-organ/inter-organ perfusion and microcirculatory dysfunction are quite heterogeneous and difficult to diagnose in time. Therefore, clinically important perioperative flow perturbations and/or oxygen supply/demand mismatches may go undetected.

We have previously shown that intraoperative sublingual microcirculatory flow is well maintained during elective major non-cardiac surgery when the mean arterial pressure (MAP) is in the range of 65–120 mmHg [2]. Nevertheless, perfusion of sublingual and other microvascular networks can be affected by various non-hemodynamic factors, such as fluid overload, reactive oxygen species, or chronic systemic inflammation [3,4,5,6]. Indeed, the perioperative setting involves multiple physiological stressors, and hemodynamic coherence with tissue oxygenation can be affected by a variety of patient-, anesthesia-, or surgery-related factors. 

In high-risk patients undergoing major surgery and critically ill patients undergoing emergency surgery, oxygen consumption (VO_2_) is commonly used to identify a shift from aerobic to anaerobic metabolism [7]. However, monitoring of intraoperative DO_2_/VO_2_ requires advanced technological equipment. Importantly, simplified equations using variables present in the arterial blood gasses that are adequately correlated with perfusion parameters may alternatively be used to monitor tissue oxygenation and metabolic alterations [8,9]. 

Although reductions in DO_2_ or higher VO_2_ can initiate complex cellular processes precipitating oxygen debt (OXD), conflicting results in clinical trials limit our ability to recommend specific monitoring and therapeutic strategies [10]. Considering that optimizing perfusion and oxygen transport to the tissues remains a complex task and that there is an unmet need for novel non-invasive techniques for continuous monitoring of DO_2_, VO_2_, and OXD in critically ill and surgical patients, we reasonably hypothesized that intraoperative changes in sublingual microcirculatory flow reflect clinically relevant transitions from aerobic to anaerobic metabolism (TRANAM). Therefore, we analyzed two prospective cohorts to assess TRANAM during elective and emergency non-cardiac surgery.

## 2. Materials and Methods

We performed an ancillary study using two prospective cohorts with high-risk patients undergoing elective major non-cardiac surgery [11] and critically ill patients undergoing emergency abdominal surgery [12]. The underlying studies were conducted in compliance with Good Clinical Practice guidelines, the Declaration of Helsinki, and relevant regulatory requirements. The original protocol (NCT03851965) was approved by the University Hospital of Larisa Institutional Review Board (IRB no. 60580, 11 December 2018). Written informed consent was obtained from each participant or their next-of-kin.

### 2.1. Study Objectives

The primary objective was to assess TRANAM during elective major non-cardiac surgery and emergency abdominal surgery. Our secondary objectives were (a) to investigate the association of TRANAM with hemodynamics and sublingual microcirculation and (b) to investigate the association of TRANAM with postoperative complications. 

### 2.2. Description of the Elective Major Non-Cardiac Surgery Cohort 

Patients of American Society of Anesthesiologists (ASA) physical status I to IV and all types of surgical approaches were eligible [11]. Exclusion criteria were infection in the last 30 days, liver disease, renal replacement therapy, allergies, inflammatory/immune disorders, asthma, obesity, mental or psychiatric disorders, alcohol abuse, connective tissue diseases, organ transplantation, steroid, antipsychotic or anti-inflammatory/immunomodulatory therapy within the previous three months, opioid therapy during the past week, and participation in another study [11].

#### Anesthetic Management

After receiving 5 mL kg^−1^ of a balanced crystalloid solution, patients were anesthetized using regimens that contained midazolam, fentanyl, ketamine, propofol, rocuronium, and a fraction of inspired oxygen of 0.7. General anesthesia was maintained using desflurane inhalation and was adjusted to maintain the patient’s Bispectral Index (BIS, Covidien, France) between 40 and 60 [13,14]. All patients were ventilated using a lung-protective strategy, while the intraoperative fraction of inspired oxygen was adjusted to maintain an arterial oxygen partial pressure between 80 and 100 mmHg. Normocapnia, normothermia, and normoglycemia were implemented throughout anesthesia [15,16,17]. Vasopressors were administered if MAP < 65 mmHg to maintain an individualized MAP level based on the preadmission levels. Balanced crystalloids were administered at a rate of 2 mL kg^−1^ h^−1^, while intraoperative blood losses were compensated by infusing balanced crystalloids (2:1 ratio) or 6% hydroxyethyl starch 130/0.4 (1:1 ratio). Packed red cells were transfused when hemoglobin concentrations were <9–10 g dL^−1^ in patients with cardiovascular comorbidities and the elderly, or <8 g dL^−1^ in those without cardiac comorbidities.

### 2.3. Description of the Emergency Surgery Cohort

We included adult patients with septic shock requiring emergency abdominal surgery. Septic shock was defined as circulatory and cellular/metabolic dysfunction that persisted despite adequate fluid resuscitation and required the administration of vasopressors [12,18]. 

#### Anesthetic Management

Before the induction of anesthesia, all patients with a central venous pressure (CVP) <4 mmHg received 7 mL kg^−1^ of a balanced crystalloid solution and the vasopressor dose was adjusted to maintain an individualized MAP level, as previously described [12]. Anesthesia was induced using regimens that contained fentanyl, ketamine, propofol, rocuronium, and a fraction of inspired oxygen of 0.7. General anesthesia was maintained using desflurane inhalation; desflurane was chosen for its stable effects on the microcirculation [19]. The depth of anesthesia was adjusted to maintain BIS between 40 and 60, and all patients were ventilated using a lung-protective strategy [12]. Normocapnia and normothermia were maintained during the perioperative period. Surgery-related blood losses were compensated by infusing balanced crystalloids (1.5:1 ratio). Packed red cells were transfused when the hemoglobin concentrations were <9–10 g dL^−1^ in subjects with cardiovascular comorbidities and the elderly, or <8 g dL^−1^ in those without cardiac comorbidities. All patients were managed using the microcirculation-guided treatment algorithm FRASK, which was applied intraoperatively following the restoration of systemic hemodynamics. End-points were the maximization of microcirculatory recruitment and the optimization of tissue oxygenation, as previously described (Figure S1) [12].

### 2.4. Measurements

#### 2.4.1. Systemic Hemodynamics

The radial artery was cannulated and connected to a FloTrac/EV1000 clinical platform (Edwards Life Sciences, Irvine, CA, USA) to measure macrohemodynamics, as previously described [12]. We directly measured the heart rate (HR), systolic arterial pressure (SAP), diastolic arterial pressure (DAP), MAP, cardiac output (CO), cardiac index (CI), stroke volume (SV), stroke volume variation (SVV), and systemic vascular resistance (SVR). In addition, the internal jugular vein was cannulated with a triple-lumen central venous catheter; this allowed the measurement of CVP and central venous oxygen saturation (ScvO_2_). The cardiac power output [CPO = (CO × MAP)/451], arterial compliance [Cart = SV/(SAP − DAP)], arterial resistance [Rart = MAP/(SV × HR)], venous compartment resistance [Rven = SVR × 0.038], and effective arterial elastance (Ea = MAP/SV) were also calculated [11,12]. 

#### 2.4.2. Calculation of Mean Circulatory Filling Pressure Analogue and Related Variables

The analogue of mean circulatory filling pressure (Pmca) was derived using the mathematical model Pmca = (a × CVP) + (b × MAP) + (c × CO), as previously described [1,11,12,20,21]. The pressure gradient for venous return (PGVR) was defined as the pressure difference between Pmca and CVP (PGVR = Pmca − CVP). The resistance to venous return (RVR) was defined as the resistance downstream of Pmca to reflect resistance to venous return and was calculated as the ratio of the pressure difference between Pmca and CVP and CO [RVR = (Pmca − CVP)/CO]. The efficiency of the heart (Eh) was defined as the ratio of the pressure difference between Pmca and CVP and Pmca [Eh = (Pmca − CVP)/Pmca] [12,20,22,23].

#### 2.4.3. Sublingual Microcirculation Analysis

Sublingual microcirculation was monitored using SDF+ imaging (Microscan; Microvision Medical BV, Amsterdam, The Netherlands), as previously described [11,12]. The first assessment was performed 30 min after the induction of general anesthesia before surgical incision. Thereafter, assessments were performed every 30 min until emergence from anesthesia or transfer to the intensive care unit. All sublingual perfusion videos were evaluated by two experienced raters blinded to all patient data and the best three videos were analyzed by a blinded investigator with AVA4.3C Research Software (Microvision Medical, Amsterdam, The Netherlands) [24,25,26]. We analyzed the De Backer score, the Consensus Proportion of Perfused Vessels (Consensus PPV), and the Consensus PPV (small).

#### 2.4.4. Oxygen Extraction and Transitions of Metabolism

The oxygen extraction ratio was calculated as the ratio of VO_2_ (VO_2_ = O_2_ER × DO_2_) to oxygen delivery {DO_2_ = CO × 10 × [(0.0138 × Hb × SaO_2_) + (0.0031 × PaO_2_)]} using the formula O_2_ER = VO_2_/DO_2_ = (SaO_2_ − ScvO_2_)/SaO_2_. Transitions from aerobic to anaerobic metabolism were monitored using OXD. The latter can be calculated at bedside using the formula described by Dunham et al., which involves the relationship between lactate and excess base [OXD = 6.322 (Lactate) − 2.311 (EB) − 9.013] [8] and demonstrates alterations in DO_2_/VO_2_ with a solid physiological basis [27]. 

### 2.5. Data Collection, Monitoring, and Management

Data analysis was based on predefined and contemporaneously recorded measurements. Data collection included demographic and morphometric characteristics, ASA physical status, risk scores, Clavien–Dindo Classification, the Comprehensive Complication Index (CCI), and anesthesia variables. This work is reported according to STROCSS criteria [28]. 

### 2.6. Statistical Analysis

Statistical analysis was performed using R v4.3. Data are presented as mean (standard deviation (SD)). A Shapiro–Wilk test was conducted to assess whether the various variables were normally distributed. Linear mixed effect (LME) models with Restricted Maximum Likelihood Estimation (REML) were used to assess the effect of OXD during surgery. LME models (rather than simple repeated-measure linear models) were used to account for varying durations of surgery between patients and the concurrent presence of repeated measurements. All models were constructed considering patients as random factors. Spearman’s rho method was used to estimate the correlation between different measurements. The Bonferroni Hochberg false discovery rate correction was applied to account for multiple comparisons. *p* values less than 0.05 were considered significant. 

## 3. Results

In total, 26 patients were included in the analysis (elective surgery, n = 13; emergency surgery, n = 13). The mean age in the elective and emergency group was 63 ± 14.4 and 70.5 ± 8.6, respectively [11,12] (Table 1). Intraoperative hemodynamics are presented in Table 2.

### 3.1. TRANAM during Elective Major Non-Cardiac Surgery

#### 3.1.1. Anesthesia Characteristics and Intraoperative Variation of Oxygen Dept

**Figure 1:**
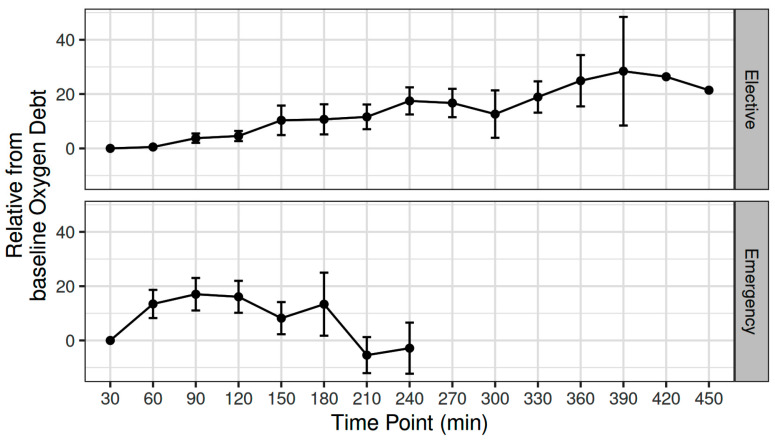
Variation in oxygen debt during elective major and emergency non-cardiac surgery.

The mean (SD) intraoperative MAP, CO, CVP, and SVR was 81.5 (6.67) mmHg, 4.6 (1) L min^−1^, 9.5 (1.79) mmHg, and 1295 (295) dynes sec cm^−5^, respectively [11]. The mean (SD) intraoperative fluid administration was 2143 (860) mL [11]. The OXD progressively increased during elective major non-cardiac surgery, with an estimated 2.24 unit increase every 30 min (adjusted *p* < 0.001) (Figure 1). 

#### 3.1.2. Association of TRANAM with Hemodynamic and Metabolic Variables

**Figure 2:**
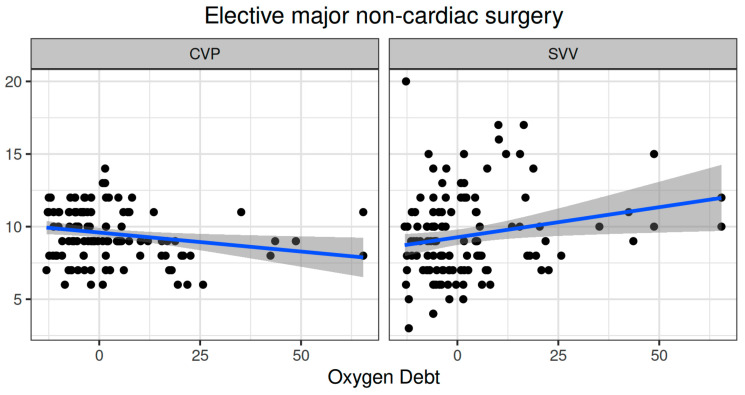
Correlation of oxygen debt with central venous pressure and stroke volume variation during elective major non-cardiac surgery. CVP, central venous pressure; SVV, stroke volume variation.

**Figure 3:**
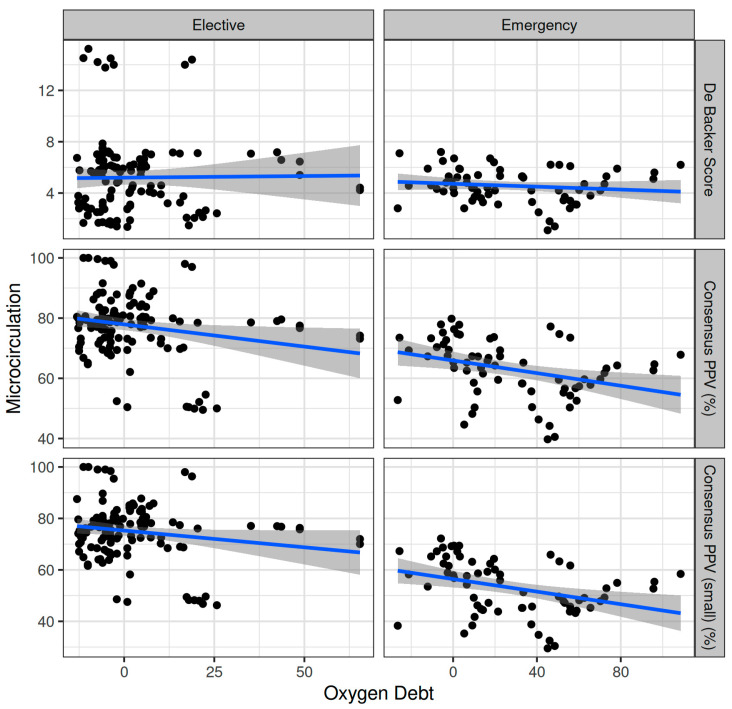
Correlation of oxygen debt with sublingual microcirculation variables during elective major and emergency non-cardiac surgery. PPV, proportion of perfused vessels.

**Figure 4:**
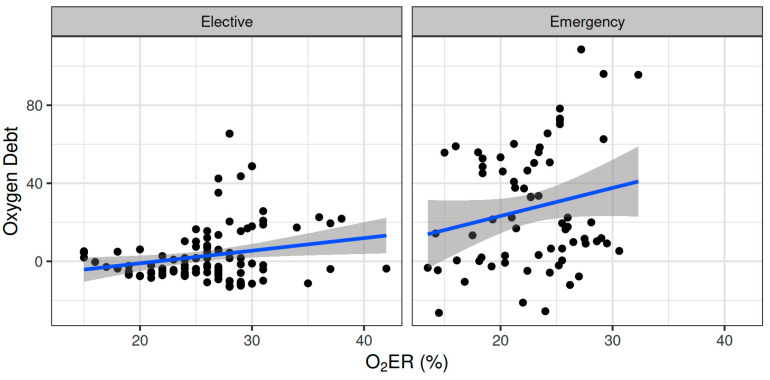
Correlation of oxygen debt with oxygen extraction ratio during elective major and emergency non-cardiac surgery. O_2_ER, oxygen extraction ratio.

Oxygen dept was negatively correlated with CVP (ρ = −0.247, adjusted *p* = 0.006) and positively correlated with SVV (ρ = 0.185, adjusted *p* = 0.041) (Figure 2). However, OXD was not significantly correlated with the sublingual De Backer score (ρ = 0.076, adjusted *p* = 0.403), Consensus PPV (ρ = −0.098, adjusted *p* = 0.281), and Consensus PPV (small) (ρ = −0.047, adjusted *p* = 0.606) (Figure 3). In addition, OXD was not significantly correlated with O_2_ER (ρ = 0.103, adjusted *p* = 0.26) (Figure 4). No significant association was observed between OXD and vasopressor use (*p* = 0.538). Table 3 depicts the association of OXD with hemodynamic and metabolic variables and the corresponding adjusted *p* values.

#### 3.1.3. Association of TRANAM with Comprehensive Complication Index

No significant correlation was observed between TRANAM and CCI in the elective major non-cardiac surgery group (ρ = 0.257, adjusted *p* = 0.581).

### 3.2. TRANAM during Emergency Abdominal Surgery

#### 3.2.1. Anesthesia Characteristics and Intraoperative Variation of Oxygen Dept

The mean (SD) intraoperative MAP, CO, CVP, and SVR were 80.3 (14) mmHg, 5.31 (0.98) L min^−1^, 9.55 (3.36) mmHg, and 995 (189) dynes sec cm^−5^, respectively. The mean intraoperative crystalloid administration was 2200 (919) mL [12]. The OXD increased during the first two intraoperative hours; thereafter, it progressively decreased during the remaining intraoperative time. When fitted as a first-degree polynomial in relation with time, i.e., if the decrease was monotonal, the variation in OXD was not statistically significant (*p* = 0.15). Nevertheless, when modelled as a second-degree polynomial, the time-squared term was statistically significant (β = −34.4, *p* = 0.017) (Figure 1). 

#### 3.2.2. Association of TRANAM with Hemodynamics and Metabolic Variables

**Figure 5:**
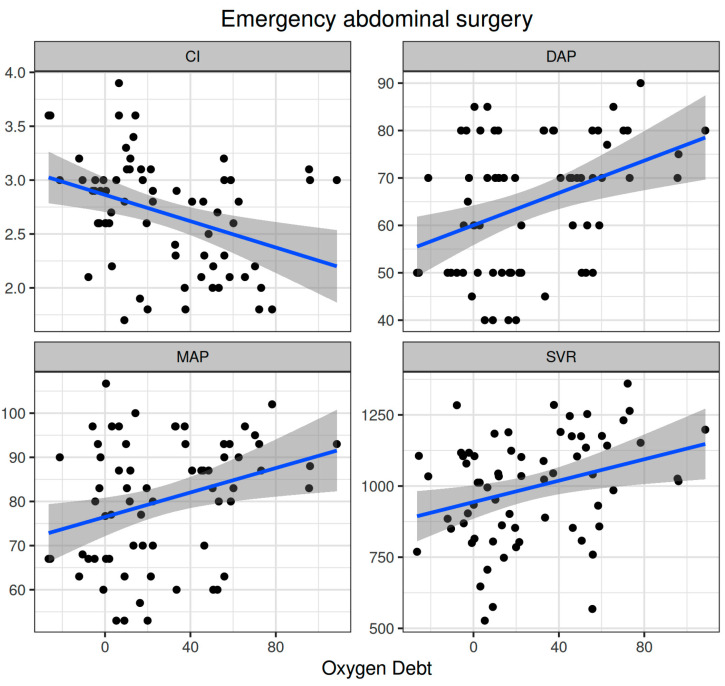
Correlation of oxygen debt with macrohemodynamic variables during emergency non-cardiac surgery. CI, cardiac index; DAP, diastolic arterial pressure; MAP, mean arterial pressure; SVR, systemic vascular resistance.

Oxygen dept was significantly correlated with CI (ρ = −0.352, adjusted *p* = 0.003) and DAP (ρ = 0.338, adjusted *p* = 0.005) and to a lesser extent with MAP (ρ = 0.278, adjusted *p* = 0.023) and SVR (ρ = 0.296, adjusted *p* = 0.015) (Figure 5). Oxygen debt was significantly correlated with Consensus PPV (ρ = −0.438, adjusted *p* < 0.001) and Consensus PPV (small) (ρ = −0.434, adjusted *p* < 0.001), but not with the De Backer score (ρ = −0.16, adjusted *p* = 0.196) (Figure 3). However, OXD was not significantly correlated with O_2_ER (ρ = 0.142, adjusted *p* = 0.253) (Figure 4). No significant correlation was observed between OXD and vasopressor use (*p* = 0.629). Table 4 depicts the association of OXD with hemodynamic and metabolic variables and the corresponding adjusted *p* values. 

#### 3.2.3. Association of TRANAM with Comprehensive Complication Index

No significant correlation was observed between TRANAM and CCI in the emergency surgery group (ρ = 0.060, adjusted *p* = 0.944) (Table 5).

## 4. Discussion

In this ancillary study, TRANAM was evident in both surgical cohorts. In elective major non-cardiac surgery, OXD progressively increased and was negatively correlated with CVP whilst positively correlated with SVV. However, it was not significantly correlated with sublingual microcirculation or O_2_ER. In patients undergoing emergency surgery, OXD increased during the first two intraoperative hours and then gradually decreased until the end of surgery. In that cohort, OXD was positively correlated with DAP and to a lesser extent with MAP and SVR, but not with O_2_ER. Also, OXD was negatively correlated with CI, Consensus PPV, and Consensus PPV (small). No apparent correlation was observed between TRANAM and CCI in both cohorts. 

Although tissue hypoxia and metabolic insufficiency can lead to increased mortality, in otherwise healthy surgical patient populations the need for higher DO_2_ is unclear [29]. In each patient, however, surgical stress leads to the activation of complex metabolic and hormonal responses, which result in variable oxygen requirements and VO_2_ [30,31,32]. Therefore, OXD could be used to identify changes in DO_2_/VO_2_ and the transition from aerobic to anaerobic metabolism [7].

In our elective cohort, macrohemodynamic parameters were maintained stable, but OXD progressively increased during surgery. Notably, OXD was not significantly correlated with sublingual microcirculation or O_2_ER. The most possible explanations for this finding include microcirculatory impairment in tissues other than the sublingual, increased venular and capillary pressure, and/or tissue dysoxia. As fluid filtration in the microcirculation is determined by the hydrostatic pressure and the intravascular oncotic pressure, any increase in CVP directly increases the former (assuming no change in venous resistances) and produces tissue edema [33], which may lead to microcirculatory alterations and a loss of hemodynamic coherence in local and adjacent networks [34]. In our study, OXD was negatively correlated with CVP whilst positively with SVV. Although intraoperative fluid administration was 2143 ± 860 mL, which is in agreement with a recommended risk-adapted fluid strategy aiming at a moderately liberal approach (with a positive fluid balance of 1–2 L at the end of surgery) [35], occult hypovolemia may occur in up to 60% of patients undergoing major surgery [36]. Also, an increased SVV may sometimes imply anesthesia-induced vasodilation, leading to unnecessary fluid administration (rather than vasopressor use) and fluid accumulation. 

In contrast to sublingual blood flow, the impact of increased CVP is likely greater in encapsulated organs in which increased parenchymal volume tamponades blood flow [33]. Furthermore, as anesthesia reduces both VO_2_ and O_2_ER [30,31,32,37,38], even the commonly used intraoperative fractions of inspired oxygen (e.g., 0.3–0.5) can lead to local hyperoxemia, which can be detrimental as a result of its systemic vasoconstrictor effect, its associated reduction in cardiac output, and/or its involvement in inducing severe inflammation in several tissue beds [39]. All these may induce or aggravate intraoperative TRANAM and organ injury, which can be evident even during the immediate and early postoperative period [34]. 

Accurate assessment and treatment of DO_2_/VO_2_ imbalances is also critical for optimizing outcomes in critically ill patients. For example, hypoperfusion decreases DO_2_, stimulating anaerobic metabolism and creating OXD [10]. Although increasing DO_2_ is a key therapeutic intervention of shock management, it remains uncertain whether it improves clinical outcomes. Apart from the fact that many tissues function normally at oxygen levels equivalent to an atmosphere of 5% oxygen and some at levels as low as 1% [1,40], high DO_2_ may indeed increase mortality as various physico-chemical factors affect the availability of free oxygen to the tissues, e.g., sepsis. Even in cases of adequate oxygen transport to tissues, the inability of the latter to take advantage of the total amount of available oxygen offered may lead to paradoxical local hyperoxemia with the above-mentioned consequences.

In our emergency cohort, severe sepsis and septic shock resulted in tissue hypoxia/dysoxia and anaerobic metabolism. Of note, OXD increased during the first two intraoperative hours and then gradually decreased until the end of surgery. During the latter, OXD was positively correlated with DAP and to a lesser extent with MAP and SVR, but not with O_2_ER. Also, OXD was negatively correlated with CI, Consensus PPV, and Consensus PPV (small). A possible explanation for these correlations is that O_2_ER had reached its critical point (i.e., the maximum O_2_ER) and was supply-dependent. Another reason may be abnormal tissue oxygen utilization and metabolism (dysoxia), which in combination with the rapid passage of red blood cells through the capillaries (hyperdynamic circulation) contributed to at least a partial dependence of tissue oxygenation on blood flow. Despite the fact that each tissue/organ has its own critical DO_2_ (the higher the O_2_ER for a given tissue, the greater the dependence on DO_2_), OXD gradually decreased until the end of surgery—in contrast to the elective patient group. This finding can be explained by the use of the novel FRASK microcirculation-guided resuscitation strategy, which improves microvascular flow and hemodynamic coherence (Figure S1) [12]. 

**Figure 6:**
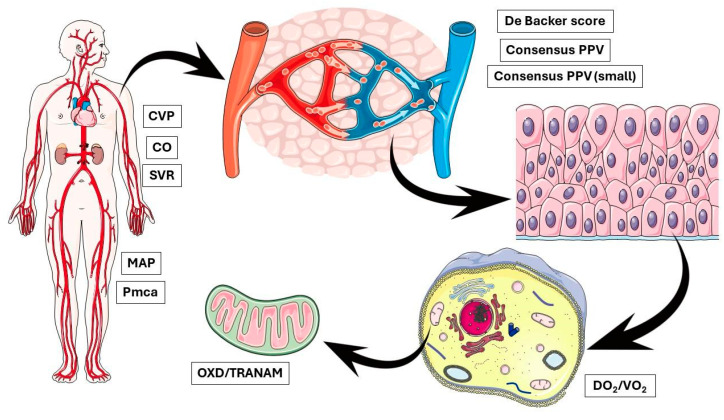
OXD and TRANAM may occur due to abnormal alterations at multiple biological levels simultaneously, indicating the existence of modifiable biological networks that generate clinical phenotypes. CVP, central venous pressure; CO, cardiac output; SVR, systemic vascular resistance; MAP, mean arterial pressure; Pmca, mean circulatory filling pressure analogue; PPV, proportion of perfused vessels; DO_2_, oxygen delivery; VO_2_, oxygen consumption; OXD, oxygen debt; TRANAM, transition from aerobic to anaerobic metabolism.

In the present study, no apparent correlation was observed between OXD and CCI in both groups. We acknowledge the small sample size of the present study that limits the interpretation of this finding and highlights the need for further evaluations of TRANAM in larger studies with heterogeneous populations. On the other hand, our findings raise important questions regarding the possible causes of OXD, especially in the elective group. This discussion is further stimulated by recent evidence suggesting that intraoperative hypotension seems to be a marker of the severity rather than a mediator of postoperative complications [41,42]. In any case, it is important to remember that physiological interactions at the molecular/cellular level are very complex. For example, capillaries are known to adapt their radii to maintain the shear stress of blood flow at the vessel wall at a set point, highlighting the potential for mechanotransduction to generate stable hydraulically tuned microvascular networks and maintain homeostasis [43,44]. In contrast, several intracellular phenomena may take place independently of the microcirculatory flow. Although microcirculatory failure, even if transiently, may lead to mitochondrial dysfunction, sustained changes in cellular metabolic activity can occur independently of macro- and microcirculatory impairment [45,46] (Figure 6). Our findings indicate the existence of modifiable biological networks that generate clinical phenotypes. Such networks could be identified by multi-level approaches integrating clinical, cellular, and molecular data.

In addition to the inherent limitations of observational studies, it is important to emphasize the single-centre origin and small sample size of the present study. we also excluded patients with conditions that might impair microcirculatory flow. However, this increases the robustness of our findings. This study was performed in a single academic department, in which expertise on cardiovascular dynamics and individualized, physiology-guided management has increased significantly over the last five years. Thus, intraoperative management may not be representative of other centres. While anesthetics can possibly impact microcirculation, the extent to which they affected the results of this study is unknown. Here, we used desflurane for maintenance due to the stable effects on the microcirculation [19]. In order to avoid or minimize the iatrogenic effects on microvascular perfusion, normoxia, normocapnia, normoglycemia, and normothermia were also maintained throughout surgery [15,16,17]. 

## 5. Conclusions

Transitions from aerobic to anaerobic metabolism were evident in both the elective major and emergency non-cardiac surgery cohorts independent of underlying alterations in the sublingual microcirculation. Clinical management and research on intraoperative OXD and TRANAM should focus on the physiological mechanisms, regulatory functions, and abnormal alterations at all biological levels, ranging from the whole organism, systems, organs, and tissues to the cellular and molecular level, as well as on their complex interactions and integration. 

## Figures and Tables

**Table 1:** Demographic and clinical characteristics of patients.

	Elective Surgery (n = 13)	Emergency Surgery (n = 13)
Age (years), median (IQR)	66 (62–72)	72 (63–76)
Sex (male), n (%)	7 (53.8)	9 (69.2)
ASA class, n (%)		
II	1 (7.7)	0 (0)
III	8 (61.5)	0 (0)
IV	4 (30.8)	0 (0)
IVE	0 (0)	13 (100)
Type of surgery, n (%)		
Gastrointestinal	9 (69.2)	9 (69.2)
Hepatobiliary	0 (0)	2 (15.4)
Urological	2 (15.4)	1 (7.7)
Vascular	2 (15.4)	0 (0)
Gastrointestinal/Vascular	0 (0)	1 (7.7)
Comorbidities		
Arterial hypertension, n (%)	6 (46.2)	10 (77)
Hypercholesterolemia, n (%)	5 (38.5)	8 (61.5)
Diabetes, n (%)	2 (15.4)	6 (46.2)
Stroke, n (%)	1 (7.7)	3 (23.1)
COPD, n (%)	1 (7.7)	4 (30.8)
Ischemic heart disease, n (%)	0 (0)	5 (38.5)
Medication		
Beta blocker, n (%)	4 (30.8)	8 (61.5)
Diuretic, n (%)	3 (23.1)	4 (30.8)
Antithrombotic, n (%)	0 (0)	10 (77)
ACE inhibitor, n (%)	0 (0)	8 (61.5)
Other, (%)	7 (53.8)	0 (0)

COPD, chronic obstructive pulmonary disease; ACE, angiotensin-converting enzyme.

**Table 2:** Intraoperative hemodynamic variables in both cohorts.

Variable	Elective Surgery	Emergency Surgery
Cardiac output (L min^−1^)	4.61 (1)	5.31 (0.98)
Cardiac index (L min^−1^ m^−2^)	2.32 (0.53)	2.7 (0.52)
Stroke volume variation (%)	9.38 (2.98)	9.49 (3.73)
Systolic arterial pressure (mmHg)	116.4 (10.66)	111.69 (15.71)
Diastolic arterial pressure (mmHg)	64.06 (6.22)	64.66 (13.88)
Mean arterial pressure (mmHg)	81.51 (6.67)	80.26 (14)
Systemic vascular resistance (dynes s cm^−5^)	1295 (294)	995 (188)
Central venous pressure (mmHg)	9.52 (1.78)	9.55 (3.35)
Mean circulatory filling pressure analogue (mmHg)	16.1 (2.18)	15.1 (4.99)
Pressure gradient for venous return (mmHg)	6.57 (1)	6 (2.39)
Resistance to venous return (mmHg min^−1^ L^−1^)	1.45 (0.21)	1.19 (0.36)
Efficiency of the heart	0.41 (0.05)	0.44 (0.11)
De Backer score (mm^−1^)	5.2 (3.03)	4.59 (1.33)
Consensus PPV (%)	77.63 (10.8)	63.06 (9.64)
Consensus PPV (small) (%)	74.94 (11.28)	53.11 (10.67)

PPV, proportion of perfused vessels. Data are presented as mean (SD).

**Table 3:** Association of oxygen depth with hemodynamic and metabolic variables during elective major non-cardiac surgery.

Variable	Rho	Adjusted *p* Value
Cardiac output (L min^−1^)	0.01 (−0.168–0.188)	0.912
Cardiac index (L min^−1^ m^−2^)	0.075 (−0.105–0.249)	0.414
Stroke volume variation (%)	0.185 (0.007–0.351)	0.041
Systolic arterial pressure (mmHg)	−0.154 (−0.322–0.025)	0.091
Diastolic arterial pressure (mmHg)	0.044 (−0.135–0.22)	0.631
Mean arterial pressure (mmHg)	−0.049 (−0.225–0.13)	0.592
Systemic vascular resistance (dynes s cm^−5^)	0.0147 (−0.0431–0.0238)	0.872
Central venous pressure (mmHg)	−0.247 (−0.407–−0.072)	0.006
Mean circulatory filling pressure analogue (mmHg)	−0.153 (−0.322–0.025)	0.091
Pressure gradient for venous return (mmHg)	0.069 (−0.11–0.244)	0.448
Resistance to venous return (mmHg min^−1^ L^−1^)	0.052 (−0.127–0.227)	0.573
Efficiency of the heart (mmHg)	0.268 (0.095–0.426)	0.002
De Backer score (mm^−1^)	0.076 (−0.103–0.251)	0.403
Consensus PPV (%)	−0.098 (−0.271–0.081)	0.281
Consensus PPV (small) (%)	−0.047 (−0.223–0.132)	0.606
End-tidal carbon dioxide (mmHg)	−0.285 (−0.44–−0.113)	0.001
Peripheral capillary oxygen saturation (%)	0.163 (−0.015–0.331)	0.072
Arterial oxygen saturation (%)	0.034 (−0.145–0.21)	0.713
Central venous oxygen saturation (%)	−0.092 (−0.265–0.087)	0.313
Arterial partial pressure of oxygen (mmHg)	0.013 (−0.165–0.191)	0.883
Arterial partial pressure of carbon dioxide (mmHg)	−0.492 (−0.616–−0.344)	<0.001
Bicarbonate	−0.778 (−0.84–−0.697)	<0.001
Base deficit (mmol L^−1^)	−0.939 (−0.957–−0.913)	<0.001
Oxygen extraction ratio (%)	0.103 (−0.077–0.275)	0.26
Venous-to-arterial carbon dioxide difference (mmHg)	0.437 (0.281–0.57)	<0.001

PPV, proportion of perfused vessels.

**Table 4:** Association of oxygen depth with hemodynamic and metabolic variables during emergency non-cardiac surgery.

Variable	Rho	Adjusted *p* Value
Cardiac output (L min^−1^)	−0.235 (−0.45–0.005)	0.055
Cardiac index (L min^−1^ m^−2^)	−0.352 (−0.546–−0.123)	0.003
Stroke volume variation (%)	−0.056 (−0.292–0.187)	0.561
Systolic arterial pressure (mmHg)	0.159 (−0.084–0.385)	0.198
Diastolic arterial pressure (mmHg)	0.338 (0.106–0.534)	0.005
Mean arterial pressure (mmHg)	0.278 (0.041–0.486)	0.023
Systemic vascular resistance (dynes s cm^−5^)	0.296 (0.06–0.501)	0.015
Central venous pressure (mmHg)	−0.049 (−0.286–0.194)	0.694
Mean circulatory filling pressure analogue (mmHg)	−0.113 (−0.344–0.131)	0.362
Pressure gradient for venous return (mmHg)	−0.036 (−0.274–0.206)	0.771
Resistance to venous return (mmHg min^−1^ L^−1^)	0.22 (−0.021–0.437)	0.074
Efficiency of the heart (mmHg)	0.010 (−0.042–0.019)	0.94
De Backer score (mm^−1^)	−0.16 (−0.385–0.084)	0.196
Consensus PPV (%)	−0.438 (−0.613–−0.221)	<0.001
Consensus PPV (small) (%)	−0.434 (−0.61–−0.216)	<0.001
End-tidal carbon dioxide (mmHg)	−0.28 (−0.487–−0.043)	0.022
Peripheral capillary oxygen saturation (%)	−0.087 (−0.32–0.156)	0.484
Arterial oxygen saturation (%)	0.253 (0.014–0.465)	0.039
Central venous oxygen saturation (%)	−0.127 (−0.356–0.117)	0.306
Arterial partial pressure of oxygen (mmHg)	0.224 (−0.018–0.44)	0.069
Arterial partial pressure of carbon dioxide (mmHg)	−0.243 (−0.457–−0.003)	0.047
Bicarbonate	−0.639 (−0.762–−0.471)	<0.001
Base deficit (mmol L^−1^)	−0.9 (−0.938–−0.842)	<0.001
Oxygen extraction ratio (%)	0.142 (−0.102–0.369)	0.253
Venous-to-arterial carbon dioxide difference (mmHg)	0.215 (−0.027–0.433)	0.081

PPV, proportion of perfused vessels.

**Table 5:** Postoperative complications in both cohorts.

	Elective Surgery (n = 13)	Emergency Surgery (n = 13)
Respiratory failure, n (%)	1 (7.7)	2 (15.4)
Hemorrhage, n (%)	1 (7.7)	1 (7.7)
Acute kidney injury, n (%)	0 (0)	1 (7.7)
Pneumonia, n (%)	0 (0)	1 (7.7)
Acute coronary syndrome, n (%)	0 (0)	1 (7.7)
Re-operation, n (%)	1 (7.7)	2 (15.4)

## Data Availability

Data can be made available upon request after publication through a collaborative process. Researchers should provide a methodically sound proposal with specific objectives in an approval proposal. Please contact the corresponding author for additional information.

## Supplementary Materials

The following supporting information can be downloaded at: https://www.mdpi.com/article/10.3390/biomedicines12081754/s1, Figure S1: The FRASK microcirculation-guided treatment strategy. i.v., intravenous; ScvO_2_, central venous oxygen saturation; PLR, passive leg raising and if not possible Trendelenburg position at 30°; RBC, red blood cell; O_2_ER, oxygen extraction ratio; CO, cardiac output. ^a^ i.v. infusion of furosemide at 0.1 mg kg^−1^ h^−1^. ^b^ A positive test was defined as a 10% increase in stroke volume after 1–1.5 min. From [12].
