# Supplementary figures and images for: Transitions from Aerobic to Anaerobic Metabolism and Oxygen Debt during Elective Major and Emergency Non-Cardiac Surgery
Source: Biomedicines. 2024 Aug 5;12(8):1754. doi: 10.3390/biomedicines12081754 (PMC11351305; doi:10.3390/biomedicines12081754)

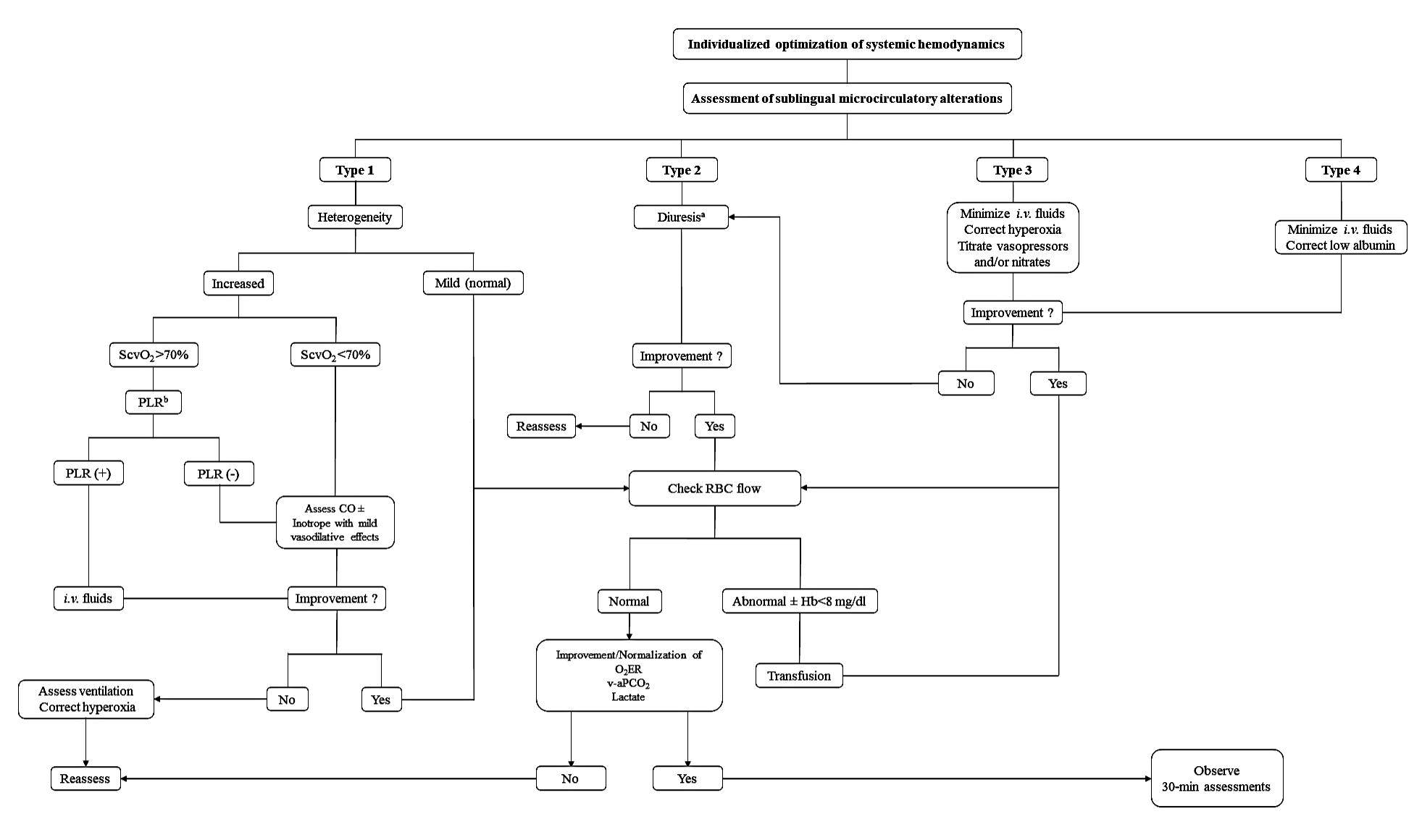

Supplement: Supplementary file 1 [file biomedicines-12-01754-s001.zip › biomedicines-3042129-supplementary.tif]
